# A novel *Pyrococcus furiosus* argonaute-based method for rapid and sensitive detection of *Mycoplasma pneumoniae* and a macrolide-resistance-related mutation

**DOI:** 10.1128/jcm.01089-25

**Published:** 2025-12-18

**Authors:** Yun Zhang, Chunhui Huo, Tao Zhang, Qian Liu, Ping He, Jiansen Du, Dongyan Xiong, Hongping Wei, Junping Yu

**Affiliations:** 1 School of Medical Technology, Xinxiang Medical University 91593, Xinxiang, PR China; 2 CAS Key Laboratory of Special Pathogens and Biosafety, Center for Emerging Infectious Diseases, Wuhan Institute of Virology, Chinese Academy of Sciences 74614 , Wuhan, PR China; 3 Qingdao International Travel Health care Center, Qingdao Customs, Qingdao, PR China; 4 University of Chinese Academy of Sciences 74519 , Beijing, PR China; Cleveland Clinic, Cleveland, Ohio, USA

**Keywords:** *Mycoplasma pneumoniae*, macrolide-resistant, PCR, PfAgo, A2063G

## Abstract

*Mycoplasma pneumoniae* (MP) is a major cause of acute respiratory diseases. While macrolide resistance in MP has been recognized for decades, the recent surge in its prevalence has emerged as a growing clinical and public health concern. Rapid, sensitive, and specific detection of MP and macrolide-resistant *Mycoplasma pneumoniae* (MRMP) are crucial for preventing the spread of the disease. By leveraging the precise programmable recognition and cleavage capabilities of *Pyrococcus furiosus* Argonaute nuclease (PfAgo), we have established a short-PCR- based method that targets both the RepMP1 gene and the A2063G mutation in the 23S rRNA, enabling efficient detection of MP and MRMP. The high GC content around the 2063 site in the 23S rRNA complicates primer and PfAgo guide DNA (gDNA) design, posing challenges to the differentiation between mutant and wild-type strains. Through meticulous design and screening of PfAgo gDNA, along with optimization of reaction conditions, we achieved a high sensitivity, where the method detects as few as 0.5 copies per reaction for MP, 5 copies per reaction for the A2063G mutation, and 14 copies per reaction for wild-type MP. The entire detection procedure can be completed in 53 min using either extracted DNA or extraction-free oropharyngeal swab samples, with no cross-reaction with the tested eight common pathogens. This methodology has been successfully applied to the detection of MP and MRMP in 58 oropharyngeal swabs, providing robust support for epidemic control of MP and offering critical guidance for the appropriate treatment of MRMP cases.

**IMPORTANCE:**

The PCR-PfAgo system developed in this study establishes a rapid, sensitive, and specific detection platform that is crucial for early identification of *Mycoplasma pneumoniae* and the macrolide resistance-associated A2063G mutation, thereby guiding clinical treatment and controlling the spread of resistant strains. With advantages including enhanced sensitivity and superior specificity, the system accurately discriminates between resistant and sensitive strains, providing critical guidance for the timely and appropriate use of antibiotics in respiratory infections. This work demonstrates the feasibility of Argonaute protein-based systems for clinical diagnostics and provides a scalable framework for detecting other pathogens and resistance markers, laying the groundwork for future developments in multiplex detection, instrument-free readout, and prospective clinical validation.

## INTRODUCTION

*Mycoplasma pneumoniae* (MP) is a major cause of community-acquired pneumonia (CAP) in both children and adults, accounting for approximately 10%–40% of all CAP cases (1). MP primarily causes lower respiratory tract infections, often referred to as “walking pneumonia” (2). Although clinical manifestations of MP infection are generally mild and self-limiting, they can also induce severe complications such as acute respiratory syndrome, acute myocardial injury, autoimmune diseases, and encephalitis, occasionally leading to death (3, 4). MP lacks a cell wall and, thus, exhibits intrinsic resistance to antibiotics targeting cell wall synthesis, such as β-lactams. Hence, treatment for MP relies mainly on antibiotics that act on bacterial ribosomes to inhibit protein synthesis (e.g., macrolides and tetracyclines) and antibiotics that inhibit DNA replication (e.g., fluoroquinolones) (5). Among them, macrolides have been the first-choice drugs for treating pediatric MP infections due to their mild adverse reactions and ease of use (6). However, due to the widespread use of the antibiotics, the prevalence of macrolide-resistant *Mycoplasma pneumoniae* (MRMP) strains has increased globally since 2000 (7). In North America and Europe, the resistance rate has reached 25%, while in some regions of Asia, it has exceeded 90% (8). The mechanism of resistance primarily stems from the mutations in domain V of the 23S rRNA gene of MP, which decrease the affinity of macrolides for the ribosome, leading to resistance (9). Among the mutations associated with MRMP infections, A2063G is the most common mutation (96.8%) (10). The rapid spread of MRMP could undermine decades of progress in managing MP infections, leading to more severe disease outcomes, longer hospital stays, and higher medical costs (11). Early and rapid detection of MP and MRMP is crucial for guiding clinical treatment to control MRMP infections.

MP culture is the diagnostic gold standard recommended by the World Health Organization, but it is time-consuming (2–6 weeks) and labor-intensive, limiting its value for early diagnosis (12). Serological testing is widely used due to its ease of use, but test results are affected by disease progression and are often retrospective (13). PCR (Polymerase Chain Reaction) is considered the new “gold standard” for detecting MP due to its high sensitivity and specificity and is widely used in clinical settings (14). Unlike antigen and antibody testing, nucleic acid testing directly identifies genetic material, providing the most direct means of detecting the presence of the target.

For the detection of MRMP, available methods are limited and primarily include targeted next-generation sequencing (NGS) technology, high-resolution melting curve analysis, and fluorescent probe techniques (15–17). Current research on MRMP predominantly focuses on mutation sites, distribution regions, clinical characteristics, and related aspects (18). Among these, sequencing-based approaches are most commonly employed for mutation detection. We speculate that this preference may be attributed to the high GC content near the major mutation site at position 2063, which poses significant challenges for alternative methods, such as Amplification Refractory Mutation System-PCR (ARMS-PCR), in detecting single-nucleotide mutations.

Over the past decade, the Argonaute nuclease from *Pyrococcus furiosus* (PfAgo) has witnessed the application in molecular detection (19–21). PfAgo is a DNA-guided, programmable DNA-cleaving enzyme that exhibits high levels of activity at temperatures between 80°C and 100°C, with no special sequence restrictions for selecting target DNA cleavage sites (22). PfAgo is activated after specific matching of guide DNA (gDNA) and the target and then performs specific cleavage between the 10th and 11th nucleotides of the target (counted from the 5′ end of the aligned gDNA). PfAgo can operate without amplification steps but has low sensitivity, so it can be combined with conventional PCR, RPA (Recombinase Polymerase Amplification) or LAMP (Loop-mediated isothermal amplification) to improve sensitivity (23).

In this study, we developed a combined PCR and PfAgo method for the detection of MP targeting RepMp1 gene and MRMP targeting the key A2063G mutation in 23S rRNA (which covers 96.8% of macrolide-resistant mutations). This method achieved ultra-high sensitivity, capable of detecting as few as 0.5 copies per reaction for MP detection and enabled single-nucleotide resolution on high GC-content fragments through a novel signal discrimination approach that distinguishes between wild-type and mutant nucleotides. Two gDNAs targeting mutant and wild-type MP, respectively, were used, with fluorescence signals acquired at 8 minutes. The entire experimental procedure was completed within 1 h. This method offers a novel and reliable technical option for rapid and sensitive detection of MP, as well as for the diagnosis and treatment of MRMP infections.

## MATERIALS AND METHODS

### Materials and reagents

All the oligonucleotides used in this study were purchased from Sangon Biotech Co Ltd (Shanghai, China). The pUC 57 plasmids, which encompass the targeted domain V of the 23S rRNA gene (both wild-type and mutant) and RepMp1, respectively, were synthesized from GeneCreate Inc. (Wuhan, China). Both PCR primers, designed to amplify the corresponding fragments, and multiple gDNA sets were developed to target the aforementioned genes. The gDNA mentioned in this study is all phosphorylated at the 5′ end. All chemicals were selected from analytical grade materials and obtained from local commercial suppliers unless otherwise stated. The DEPC-treated water was used in this experiment. All buffers were prepared using deionized water with a resistivity of 18.2 MΩ.

### Bacterial strains and clinical samples

Several common respiratory pathogens, including Human adenovirus-1 (BNCC 363161), Human adenovirus-2 (BNCC 364678), Human adenovirus-5 (BNCC 363890), as well as *Haemophilus influenzae* (ATCC 49766), *Streptococcus pneumoniae* (ATCC 49619), *Klebsiella pneumoniae* (ATCC 13883), and *Mycoplasma pneumoniae* (ATCC 15531), have been preserved in our laboratory. Bacterial suspensions of different concentrations of the pathogens were spiked into negative oropharyngeal swab samples to prepare simulated samples. Twenty-three clinical MP-positive oropharyngeal swab samples, 17 clinical MP-negative swab samples, and 2 *ureaplasma urealyticum*-positive samples used in this study were provided by the Third Affiliated Hospital of Xinxiang Medical University. The use of human oropharyngeal swab samples in this study was approved by the Ethics Committee of Xinxiang Medical University (No. XYLL-2021044). All samples were de-identified residual specimens remaining after completion of routine clinical diagnostics. In accordance with national and international guidelines for research involving residual clinical specimens, the Ethics Committee waived the requirement for informed consent given the non-interventional nature of the study and the absence of personal identifiers. Nucleic acids were extracted from the oropharyngeal swab samples using the QIAamp DNA Mini Kit (Qiagen, Germany).

### Fast PCR

A fast PCR system consisted of a total volume of 20 µL, including 0.6 µL each of 10 µM forward and reverse primers, 10 µL of 2 × AceQ Mix (Q112, Vazyme, Nanjing, China), 3.8 µL of nuclease-free water, and 5 µL of template. The template was a pUC 57 plasmid containing either a 98 bp fragment of domain V of the 23S rRNA gene or an 87 bp sequence of the RepMp1 gene, the sequences of which were shown in Supplementary materials (Table S1). The fast PCR amplification conditions were as follows: an initial denaturation at 95°C for 5 min, followed by 40 cycles of denaturation at 95°C for 10 s, annealing and extension at 57°C for 10 s. The whole PCR procedure was completed within 45 min. The PCR products were subsequently verified using a 3% agarose gel.

### PfAgo cleavage assay

The PfAgo protein was expressed and purified and demonstrated high cleavage activity against target sequences using gDNA, according to the methods described in our previous publication (23). The PfAgo cleavage system consisted of a 20 µL volume, containing 2 µL of 10 × PfAgo reaction buffer, 0–10 µM of PfAgo, 0–8 mM of MnCl_2_, 0–2 µM of probe, and 0–2 µM of gDNA for optimization. The cleavage reaction was performed using a Bio-Rad CFX 96 instrument (Bio-Rad, Hercules, CA, USA) with an incubation at 95°C. Fluorescent signals were recorded at 1-min intervals over a period of 30 min to optimize the collection time.

### Urea-polyacrylamide gel electrophoresis

Urea-polyacrylamide gel electrophoresis (Urea-PAGE) was utilized for the screening of gDNA. Nucleic acid samples were mixed with 2 × RNA loading buffer (purchased from Shanghai Sangon Biotech Co., Ltd.) in a 1:1 ratio and denatured by heating at 95°C for 5 min. The denatured samples were then loaded onto precast 20% Urea-PAGE gel, where acrylamide/bis (also sourced from Shanghai Sangon Biotech Co., Ltd.) was prepared with a final concentration determined based on the length of the nucleic acid fragments to be resolved. In this experiment, a 40% Acr/Bis (29:1) solution was used. Electrophoresis was performed at a constant voltage of 150 V in 1 × TBE buffer for 1.5 h. Following electrophoresis, the gel was stained with 1 × SYBR Gold nucleic acid dye (Thermo Scientific) in the dark for 10 min to visualize the nucleic acid bands. The stained gel was subsequently imaged using a gel documentation system (ChemiDoc XRS+, BioRad, Hercules, CA, USA).

### Validation of clinical samples

To further validate the feasibility of the established PCR-PfAgo platform for field sample analysis, the platform was employed to detect MP and MRMP in clinical oropharyngeal swabs. Initially, nucleic acids were extracted from clinical oropharyngeal swab samples followed by measuring CT values using qPCR to determine the positivity of the samples to MP. Moreover, PCR and Sanger sequencing were utilized to determine the presence of the A2063G mutation in MP from the samples. Given the absence of wild-type MP strains in the clinical samples, different concentrations of wild-type MP (ATCC 15531) were spiked into various negative oropharyngeal swab samples to simulate wild-type MP samples. The qPCR reaction mixture consisted of 10 µL of 2 × AceQ Mix, 0.4 µL of each 10 µM forward and reverse primers, 0.2 µL of probe, 4 µL of nuclease-free water, and 5 µL of template DNA. The primer and probe sequences were adapted from a previous study (24) as follows, F: 5′-CGTCCWGGKATGTAYATHGG-3′, R: 5′-CCHACRCCRTGWAAWCCDCC-3′, P: ATAACTGTTAGTGACAACGGTCG. A standard curve of copy numbers vs Ct values was plotted according to ddPCR results of a serial dilution of the nucleic acid from a MP-positive sample. The droplets of the ddPCR were generated using an automated droplet generator, and signal reading was performed on QX 200 AutoDG (BioRad, USA). The ddPCR system included 10 µL of 2 × Supermix (no dUTP), 250 nM primers and probe, 5 µL of template DNA, and nuclease-free water to make a final volume of 20 µL. The primers and probe used in ddPCR were identical to those in qPCR. The ddPCR detection results and the standard curve are presented in Fig. S1 in the supplementary materials. Finally, the extracted DNA from both the clinical and spiked sample was tested using the PfAgo system. For MP and MRMP detection, fluorescence was measured 8 min post PfAgo cleavage.

### Principle of the PCR-PfAgo method

**Fig 1:**
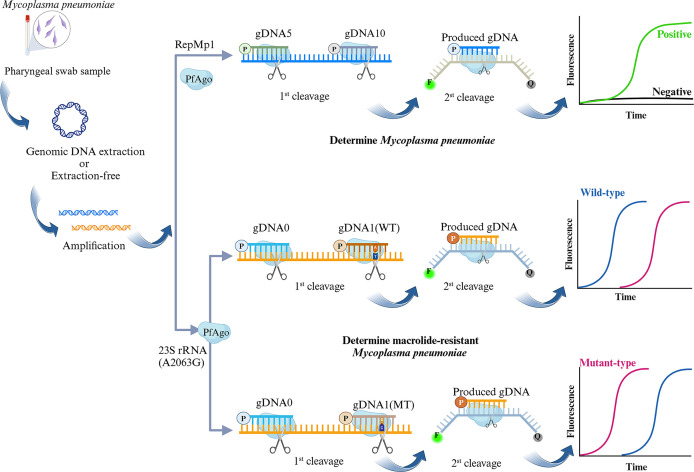
The schematic diagram of the PCR-PfAgo method for detecting MP and MRMP. Created in https://BioRender.com.

The principle of the PCR-PfAgo method is illustrated in Fig. 1. Genomic DNA can be extracted from pharyngeal swab samples containing MP. Alternatively, strongly positive samples (typically indicated in practice by a low qPCR Ct value (e.g., ≤30) may be directly used for subsequent tests without the need for extraction. In this combined assay, target regions of the genome are amplified via PCR. The amplification template sequences are provided in Table S1. The resulting amplicons are subsequently subjected to cleavage by PfAgo, guided by two gDNAs. During the base pairing between the gDNA and one strand of the amplicon, PfAgo cleaves the phosphodiester bond between the 10th and 11th nucleotides of the target complementary DNA strand from the position of the 5′-phosphorylated end of the gDNA. This initial cleavage generates a new 5′-phosphorylated single-stranded DNA (ssDNA), which subsequently serves as a secondary gDNA to guide further cleavage by PfAgo. The substrate for this secondary cleavage is a synthetically designed single-stranded DNA probe labeled at its termini with a FAM fluorophore and a BHQ-1 quencher. Upon secondary cleavage, the released FAM fluorescence signal can be detected using a fluorescence detector. For the detection of MP, positive samples exhibit a fluorescence signal, whereas negative samples do not. For the detection of MRMP, both wild-type and mutant samples produce fluorescence signals. However, they are differentiated based on the temporal difference in the appearance of the fluorescence signal.

### The selection of target amplicon fragments

For the detection of MP, a 98 bp DNA fragment from the repetitive element RepMp1 in the MP genome was selected as the target for detecting MP, as RepMp1 appears with the highest copy number (~14 copies) compared to other RepMp sequences in the MP genome (25). Moreover, the large repetitive sequence of RepMp1 is specific to MP, and similar sequences have not been found in any other sequenced mycoplasma species (26). 223 complete genomes of MP were downloaded from NCBI, and the mutation status of the RepMp1 amplicon sequence was analyzed by our developed tool PHDtools (27), revealing complete consistency to verify the conservation of this amplicon. For the detection of MRMP, a short target with an 87 bp fragment of domain V of the 23S rRNA gene containing the A2063G mutation site was selected. The length of the target fragments was determined based on the following three considerations: I. shorter amplicons reduce PCR time; II. the PfAgo detection process involves two rounds of cleavage, with the gDNA for the second cleavage being derived from the first cleavage product of the PCR amplicon; III. based on our experience, gDNA within the length range of 15–30 nt exhibits optimal activity in mediating PfAgo cleavage of ssDNA.

## RESULTS

### Screening of gDNA

**Fig 2:**
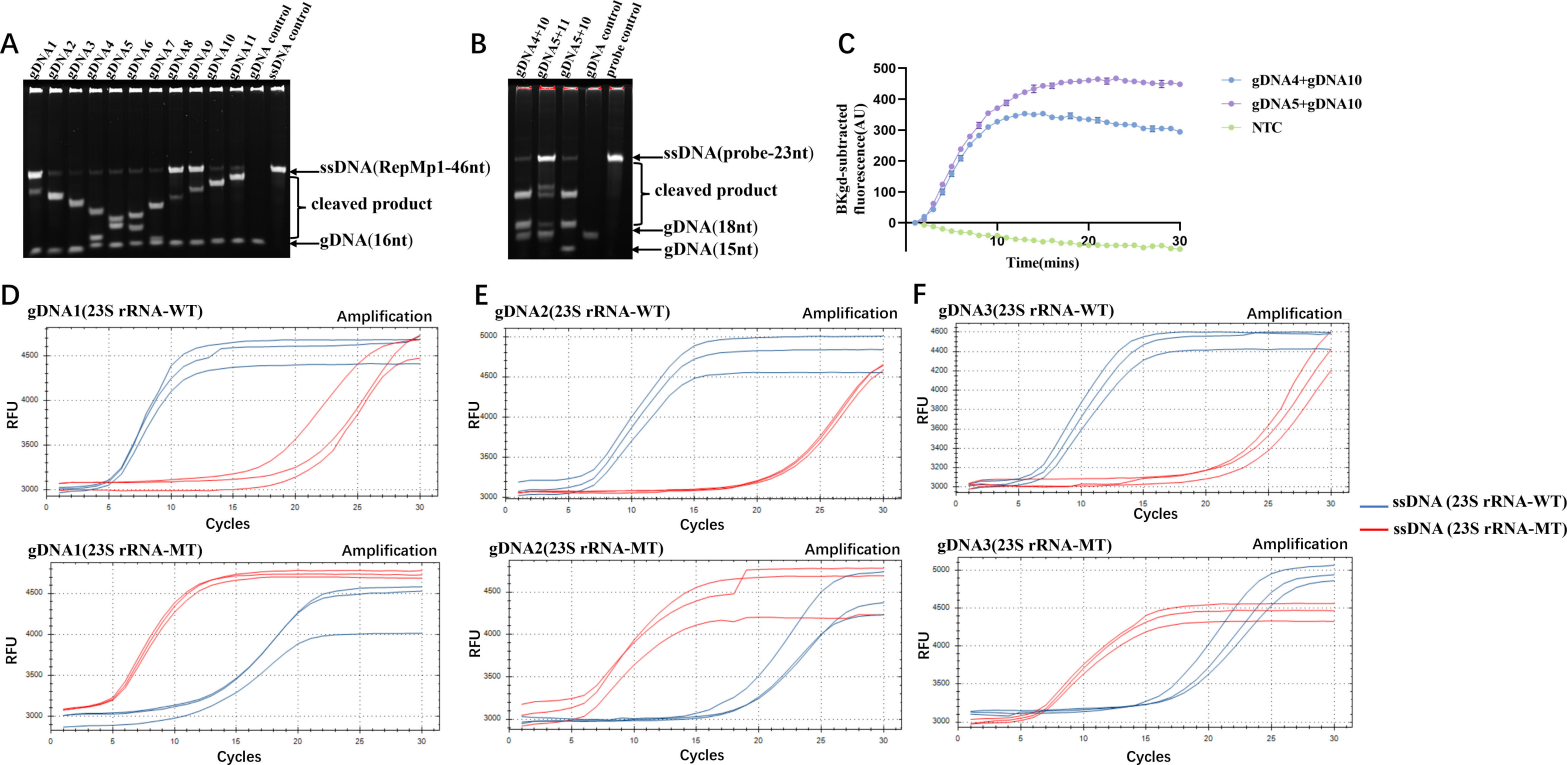
Screening of gDNA and Activity Assessment of PfAgo Protein. Figures (**A**) to (**C**) correspond to the target RepMp1 of MP, while Figures (**D**) to (**F**) focus on detecting A2063G mutation in MRMP. (**A**) Urea-PAGE gel illustrating PfAgo-mediated cleavage of ssDNA (RepMp1) templates screening the first gDNAs. Specifically, the lanes from left to right represent gDNA1 to gDNA11 at the presence of the template of ssDNA (RepMp1), the sole gDNA11 as a gDNA control and the sole ssDNA (RepMp1), respectively. (**B**) Urea-PAGE gel displaying PfAgo cleavage of ssDNA (probe) guided by secondary gDNAs. Specifically, the lanes from left to right represent ssDNA (the probe sequence) cleaved by PfAgo under the guidance of gDNA4/gDNA10, gDNA5/gDNA11, or gDNA5/gDNA10, sole gDNAs and ssDNA (probe) alone, respectively. (**C**) Fluorescent intensity over time (in minutes) of ssDNA (RepMp1) cleaved by PfAgo guided by gDNA4/gDNA10 (blue curve), and gDNA5/gDNA10 (purple curve), respectively. The green curve (NTC, negative control) shows the signal without ssDNA (RepMp1). The *y*-axis shows background-subtracted fluorescence intensity. Error bars represent the standard deviation of three replicates. Figures (**D**) to (**F**) depict the fluorescent curves over time (in minutes) of ssDNA (WT or MT) cleaved by PfAgo guided by WT gDNA (blue curves) and MT gDNA (red curves). Fluorescence signals are expressed as relative fluorescence units (RFU). (**D**) Illustrates the cleavage of ssDNA (WT) (upper panel) and ssDNA (MT) (lower panel) by PfAgo guided by gDNA1 (23S rRNA-WT) (blue curves) and gDNA1 (23S rRNA-MT) (red curves). (**E**) Shows cleavage of ssDNA (WT) (upper panel) and ssDNA (MT) (lower panel) by PfAgo guided by gDNA2 (23S rRNA-WT) (blue curves) and gDNA2 (23S rRNA-MT) (red curves). (**F**) Demonstrates the cleavage of ssDNA (WT) (upper panel) and ssDNA (MT) (lower panel) by PfAgo guided by gDNA3 (23S rRNA-WT) (blue curves) and gDNA3 (23S rRNA-MT) (red curves).

For the screening of gDNA for MP detection, 11 gDNAs were designed to evaluate the cleavage activity of the PfAgo/gDNA complex towards corresponding ssDNA. Each subsequent gDNA is shifted three bases to the right relative to the previous one, starting from gDNA1. The sequences of gDNA1 to gDNA11 are detailed in Table 1. A segment of ssDNA (as listed in Table 1) was synthesized in the middle of the PCR-amplified sequence to serve as the target for the gDNA screening process. Urea-PAGE was employed to evaluate the cleavage efficiency of PfAgo on ssDNA templates guided by the 11 gDNAs. As shown in Fig. 2A, all 11 gDNAs could direct PfAgo to effectively cleave the templates. Based on the high cleavage efficiency of the complex, the regions with multiple base mutations, and the location of the reporter gene, pairs of gDNA sequences (gDNA4 and gDNA10, or gDNA5 and gDNA11, or gDNA5 and gDNA10) were selected. Subsequently, Urea-PAGE was used to verify the cleavage efficiency of the secondary gDNAs corresponding to these three sets of gDNA sequences in directing PfAgo to cut the reporter gene, as shown in Fig. 2B. The secondary gDNAs corresponding to gDNA4 and gDNA10, as well as gDNA5 and gDNA10, demonstrated good guidance for PfAgo in cleaving the reporter gene sequence. To screen for the most suitable pair of gDNAs and verify the functionality of the system, initial tests were conducted by using gDNA4 and gDNA10, as well as gDNA5 and gDNA10, respectively, as probe combinations for detecting MP DNA. As shown in Fig. 2C, gDNA5 and gDNA10 achieved an earlier and more pronounced fluorescent signal compared to gDNA4 and gDNA10, with no fluorescent signal observed in the negative control. Ultimately, gDNA5 and gDNA10 were selected as the gDNAs for MP detection.

**TABLE 1:** The sequence used for MP detection

Name	Sequence (5′−3′)
gDNA1(RepMp1)	GTGTTCACTGGTATAA
gDNA2(RepMp1)	TTCACTGGTATAACCG
gDNA3(RepMp1)	ACTGGTATAACCGGTT
gDNA4(RepMp1)	GGTATAACCGGTTTGT
gDNA5(RepMp1)	ATAACCGGTTTGTTAA
gDNA6(RepMp1)	ACCGGTTTGTTAAGTT
gDNA7(RepMp1)	GGTTTGTTAAGTTTAA
gDNA8(RepMp1)	TTGTTAAGTTTAAATT
gDNA9(RepMp1)	TTAAGTTTAAATTGTC
gDNA10(RepMp1)	AGTTTAAATTGTCTGT
gDNA11(RepMp1)	TTAAATTGTCTGTTGA
RepMp1-forward	AACTCTTTACGCGTTACGTATTC
RepMp1-reverse	AACAACAGTGTGGAATTCTCTGG
RepMp1-probe	CGGTTTGTTAAGTTTAAATTGTC
ssDNA (probe)	CGGTTTGTTAAGTTTAAATTGTC
ssDNA (RepMp1)	TCAACAGACAATTTAAACTTAACAAACCGGTTATACCAGTGAACAC

For the screening of gDNA for specific identification of MRMP, seven sequences were designed. To fully leverage the specific recognition and cleavage properties of PfAgo for high-sensitivity genotyping of the A2063G mutation, two 59 nt ssDNA fragments (listed in Table 2) were synthesized as cleavage targets, containing either a T (wild-type, WT) or C (mutant, MT) base corresponding to the A2063G mutation. In Table 2, bold was used to highlight the nucleotide at position 2063 for clear identification of WT versus MT sequences, and bold/italics were employed to indicate the positions and types of introduced mismatches. There have been two strategies to design gDNA to detect mutations by PCR-PfAgo methods. According to a previous study (28), single-nucleotide variations in gDNA can significantly influence the cleavage efficiency of PfAgo. Therefore, we designed 10 gDNA molecules by placing G bases at positions 1 to 10 of the gDNA (sequences listed in Table S2 of the supplementary materials), ensuring precise matching with the MT sequence and single-base mismatch with the WT sequence. Urea-PAGE was used for verification, and the results showed that both MT and WT ssDNA targets were cleaved indistinguishably at 95°C (as shown in Fig. S2 in supplementary materials). This result indicated that single-nucleotide differences in gDNA do not affect its matching with the target in this study, and thus do not affect PfAgo’s cleavage activity toward the target. Another strategy is realized by introducing specific mismatches to lead to “bubbles” in the gDNA-ssDNA pairing and weaken recognition and cleavage (29). To differentiate between WT and MT sequences, we uniformly positioned the 11th base of gDNA at position 2063 and set it to G, while introducing additional mismatched nucleotides at positions 1 to 10 (sequences are listed in Table S3 in Supplementary Materials). Additionally, since gDNA has good activity in mediating PfAgo cleavage of ssDNA within the length range of 15′30 nt, we designed one gDNA (gDNA0(23S rRNA)) as the first gDNA in this study, resulting in newly generated secondary gDNA with a length of 20 nt. Urea-PAGE verification showed that MT and WT could still not be distinguished (as indicated in Fig. S3 in supplementary materials). Previous studies have shown that the base composition and sequence order near the cleavage site are crucial for the selection specificity of PfAgo (30). Specifically, when G-C base pairs are more abundant near the cleavage site, PfAgo’s selectivity tends to be poorer; whereas when A-T base pairs prevail, PfAgo’s selectivity is generally better. We also observed that the identity of mismatched nucleotides in gDNA affects PfAgo’s discrimination behavior, and three positions (7, 10, and 11) were identified as hotspots for introducing mismatches when designing gDNA (29). We found that the GC content of the 15 bases (GCTTCACGGGGTCTT[2063C]CCGTCCCGTTGCGCC) before and after the position 2063 is higher than 70%, which could be the reason why both strategies failed. Due to the inability of the two strategies to differentiate MT from WT with a single mismatch, we attempted to introduce two consecutive mismatches to enhance PfAgo’s selectivity, with the gDNA sequences detailed in Table 2. To conduct preliminary tests, these gDNA strands were combined with gDNA0(23S rRNA) and used separately with probe sets targeting both WT and MT sequences, as illustrated in Fig. 2D through F. When additional mismatches were introduced, PfAgo preferentially cleaved the ssDNA that more closely matched the gDNA. This means that gDNA corresponding to the WT template was preferentially cleaved by the gDNA1 (23S rRNA-WT). By leveraging the temporal difference in fluorescence generation, differentiation between WT and MT was achieved. Among them, gDNA1 (23S rRNA-WT) exhibited more stable and rapid effects, prompting the selection of gDNA1 (comprising gDNA1(23S rRNA-WT) and gDNA1(23S rRNA-MT)) and gDNA0(23S rRNA) as the gDNA for detecting MRMP.

**TABLE 2:** The sequence used for MRMP detection^*a*^

Name	Sequence (5′−3′)
gDNA0(23S rRNA)	TGAAGACACCCGTTAG
gDNA1(23S rRNA-WT)	AACG***AA***ACGG**A**AAGAC
gDNA1(23S rRNA-MT)	AACG***AA***ACGG**G**AAGAC
gDNA2(23S rRNA-WT)	AACGGGAC***TT*A**AAGAC
gDNA2(23S rRNA-MT)	AACGGGAC***TT*G**AAGAC
gDNA3(23S rRNA-WT)	AACGGGAC***AA*A**AAGAC
gDNA3(23S rRNA-MT)	AACGGGAC***AA*G**AAGAC
23S rRNA-forward	ATCCAGGTACGGGTGAAGAC
23S rRNA-reverse	GTCCTGATCAATATTAAGCTACAGT
23S rRNA -probe	CCCGTTAGGCGCAACGGGACGG
ssDNA (23S rRNA-WT)	GCTTCACGGGGTCTT**T**CCGTCCCGTTGCGCCTAACGGGTGTCTTCACCCGTACCTGGAT
ssDNA (23S rRNA-MT)	GCTTCACGGGGTCTT**C**CCGTCCCGTTGCGCCTAACGGGTGTCTTCACCCGTACCTGGAT

^*a*^ Bold was used to highlight the nucleotide at position 2063 for clear identification of WT versus MT sequences, and bold/italics were employed to indicate the positions and types of introduced mismatches.

### Optimization of the PCR-PfAgo

To achieve optimal performance of the PCR-PfAgo platform to determine MP and MRMP, the concentrations of PfAgo, Mn^2+^, gDNA, and reporter probe in the reaction mixture were optimized.

**Fig 3:**
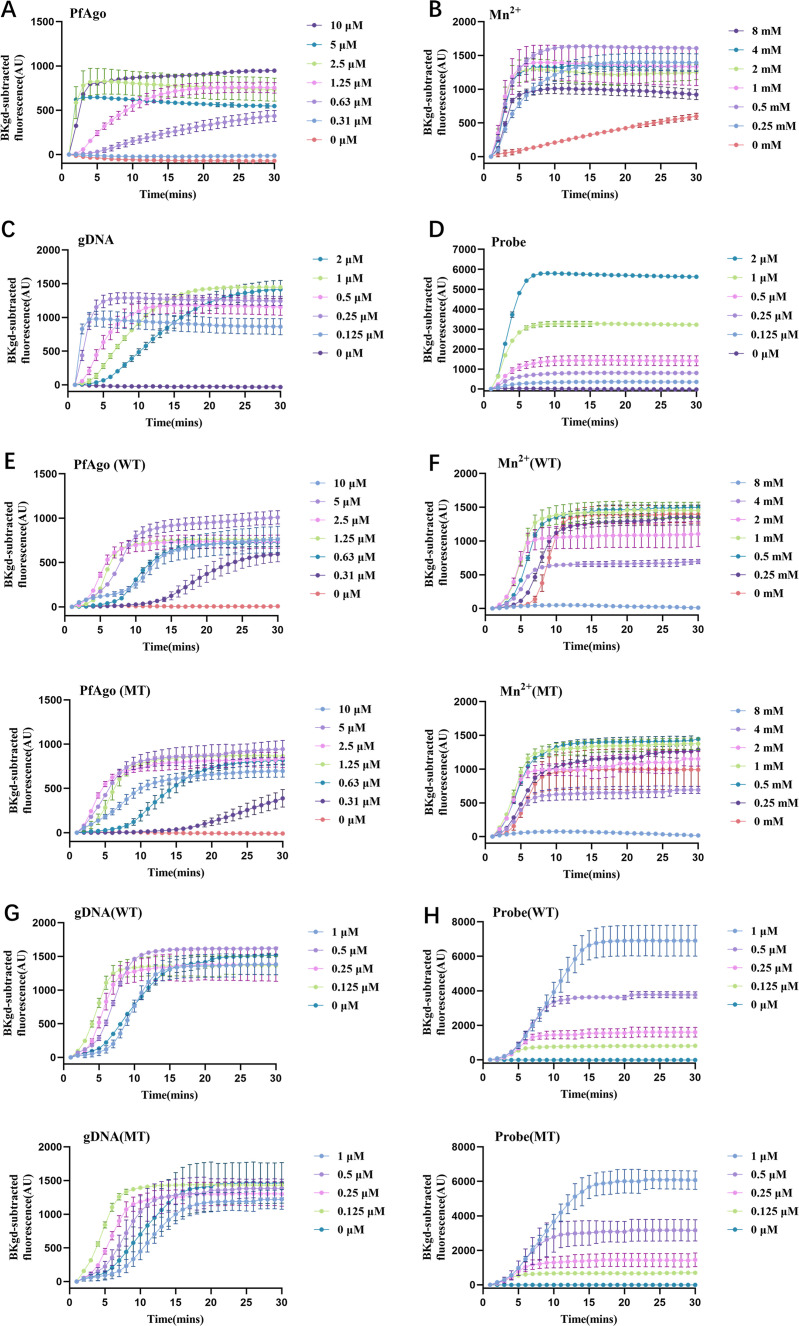
Optimization of PfAgo reaction conditions for different targets. (**A**) to (**D**) display the optimization of PfAgo reaction conditions for RepMp1. (**A**) Optimization of PfAgo concentration for RepMp1 to determine MP. (**B**) Optimization of Mn^2+^ concentration for RepMp1 to determine MP. (**C**) Optimization of gDNA concentration for RepMp1 to determine MP. (**D**) Optimization of probe concentration for RepMp1 to determine MP. (**E**) to (**H**) illustrate the optimization of PfAgo reaction conditions for A2063G to determine MRMP. (**E**) Optimization of PfAgo protein concentration for WT MP (upper panel) and MT MP (lower panel). (**F**) Optimization of Mn^2+^ concentration for WT MP (upper panel) and MT MP (lower panel). (**G**) Optimization of gDNA concentration for WT MP (upper panel) and MT MP (lower panel). (**H**) Optimization of probe concentration for WT MP (upper panel) and MT MP (lower panel). Background-subtracted fluorescence intensity is shown on the *y*-axis. Error bars represent the standard deviation of three replicate experiments.

Seven different concentrations of PfAgo, ranging from 0 to 10 µM, were tested, and the optimal PfAgo concentration was determined to be 2.5 µM based on the observed fluorescence signal (Fig. 3A). Similarly, seven different Mn^2+^ concentrations ranging from 0 to 8 mM were tested, with 1 mM identified as the optimal Mn^2+^ concentration (Fig. 3B). Six different gDNA concentrations ranging from 0 to 2 µM were tested, and the optimal gDNA concentration was determined to be 0.25 µM (Fig. 3C). Additionally, six different reporter probe concentrations ranging from 0 to 2 µM were tested, with 1 µM determined as the optimal reporter probe concentration (Fig. 3D).

The tested concentrations and optimal concentrations of PfAgo and Mn^2+^ were consistent with those optimized for detecting MP (Fig. 3E and F). Five different gDNA concentrations ranging from 0 to 1 µM were tested, and the optimal gDNA concentration was determined to be 0.125 µM (Fig. 3G). Five different reporter probe concentrations ranging from 0 to 1 µM were tested, with 0.25 µM determined as the optimal reporter probe concentration (Fig. 3H).

### Sensitivity and specificity of the PCR-PfAgo method

**Fig 4:**
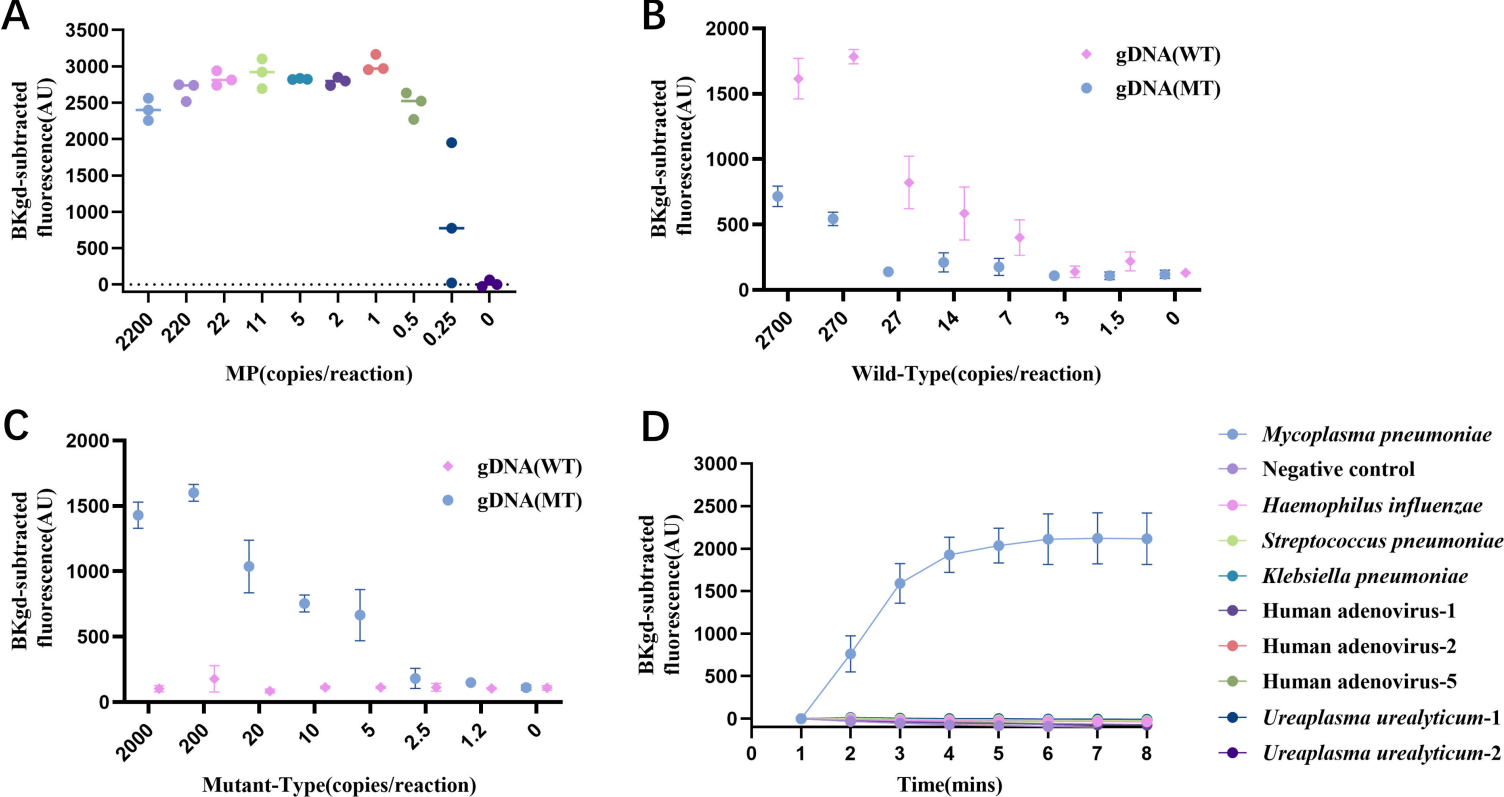
Evaluation of sensitivity and specificity of the PCR-PfAgo method. (**A**) Fluorescence values at 8 min for each dilution of MP were recorded, with three replicates for each dilution. (**B**) Fluorescence values at 8 min for each dilution of WT MP were recorded, with pink diamonds representing WT gDNA and blue dots representing MT gDNA. (**C**) Fluorescence values at 8 min for each dilution of MT MP were recorded, with pink diamonds representing WT gDNA and blue dots representing MT gDNA. (**D**) The specificity of the PCR-PfAgo method was evaluated using genomic DNA from Human adenovirus types 1, 2, and 5, as well as bacterial suspensions of *Haemophilus influenzae*, *Streptococcus pneumoniae*, *Klebsiella pneumoniae,* and *Mycoplasma pneumoniae*, as well as two cervical swab specimens positive for *Ureaplasma urealyticum*. Error bars represent the standard deviation of three replicate experiments.

To evaluate the sensitivity of the established PCR-PfAgo method, DNA from inactivated MP bacterial suspensions was used as the templates at various dilution concentrations ranging from 2 × 10^3^ to 0 copies per reaction. The fluorescent curves for all diluted DNA solutions were recorded with 1 min intervals, with the plateau of the curves appearing within 10 minutes. We monitored the fluorescence signals of mutant and wild-type samples at high, medium, and low concentration levels using two pairs of gDNAs specific to mutant and wild-type MP, respectively, and recorded the signal differences between the two detection systems over time. A graph illustrating the time-dependent changes in fluorescence differences is provided in Fig. S4. Based on the kinetic trends—particularly the point where sample discrimination was maximized across the tested concentrations while minimizing assay time—we determined 8 min as the optimal time for fluorescence signal acquisition. As shown in Fig. 4A, the detection limit of this method for MP was 0.5 copies per reaction, demonstrating a reliable advantage in sensitivity. The detection limit of this method was compared with qPCR with a commercial qPCR probe mix. The detection limit of the qPCR kit was 5 copies per reaction (see Fig. S5), indicating that the PCR-PfAgo platform had higher sensitivity. To differentiate between WT and MT MP in samples for determining MRMP, separate reactions were performed using gDNA (WT) and gDNA (MT). As shown in Fig. 4B and C, for WT MP, the system demonstrated a detection limit of 14 copies per reaction, indicating its capability to detect WT MP with high sensitivity using the designed gDNA. For MT MP, the detection limit was determined to be as few as 5 copies per reaction, highlighting the system’s ability to differentiate MT MP with high sensitivity. To evaluate the specificity of the established PCR-PfAgo method, it was applied to detect clinically common pathogens including Human adenovirus-1 (3.16 × 10^6^ TCID_50_/mL), Human adenovirus-2 (3.16 × 10^6^ TCID_50_/mL), Human adenovirus-5 (1.6 × 10^6^ TCID_50_/mL), *Haemophilus influenza* (7.5 × 10^6^ CFU/mL), *Streptococcus pneumoniae* (8 × 10^6^ CFU/mL), *Klebsiella pneumoniae* (1.3 × 10^6^ CFU/mL) and two cervical swab specimens positive for *Ureaplasma urealyticum* (*Ureaplasma urealyticum*-1 with Ct of 22.46, *Ureaplasma urealyticum*-2 with Ct of 33.78, the detection method is displayed in Table S4). The signals obtained from these non-MP pathogens were comparable to those of negative control, as indicated in Fig. 4D, demonstrating that the established method exhibits high specificity for MP.

### Examination of clinical samples

**Fig 5:**
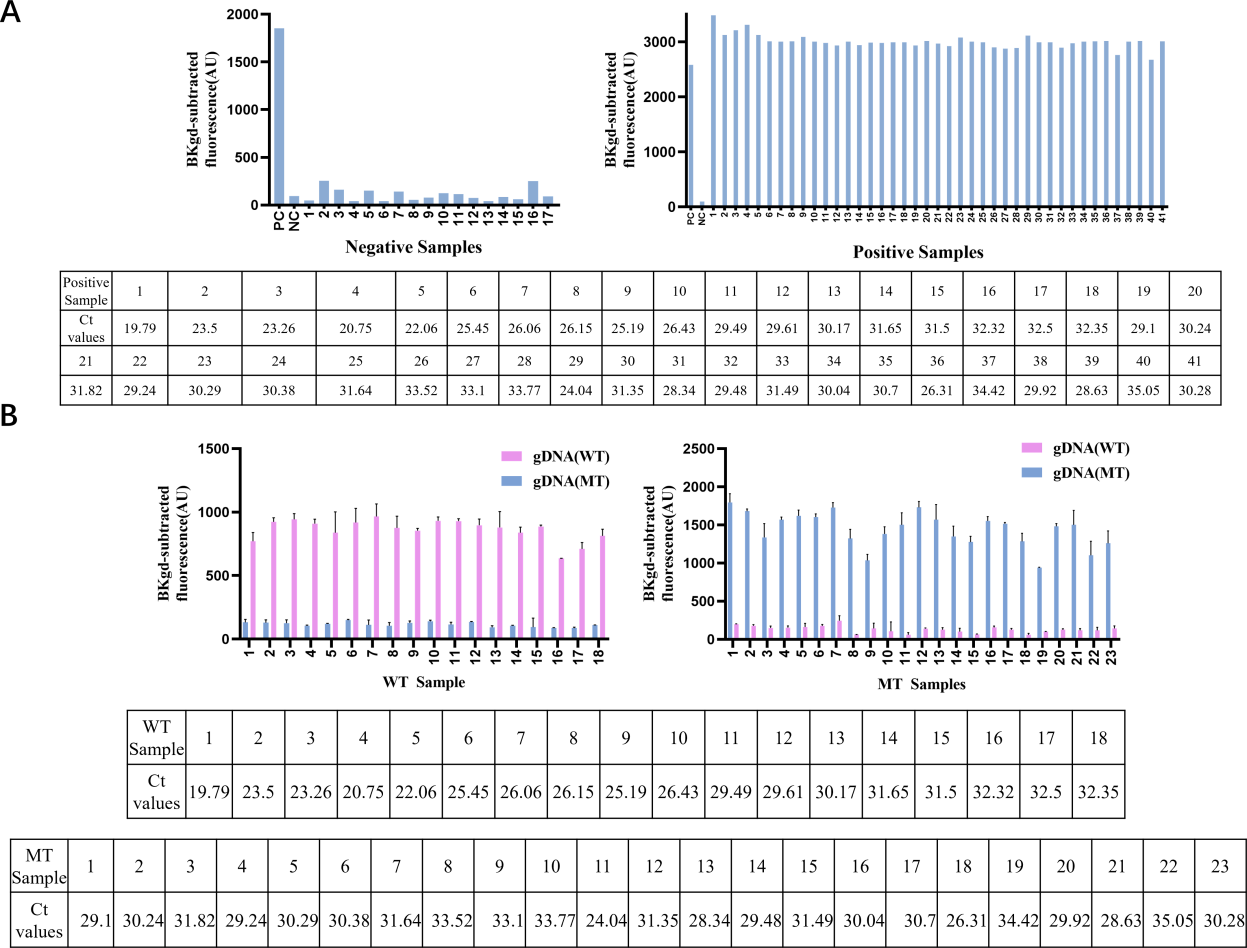
Validation of the PCR-PfAgo system using clinical samples. (**A**) The fluorescent values at 8 min recorded for each sample (the graphs) and Ct values (the table) for the 17 MP negative samples (left panel) and 41 positive samples (right panel). (**B**) The fluorescent values at 8 min by WT gDNA (pink) and MT gDNA (blue) for the 18 WT MP-spiked samples (left panel), the fluorescent values at 8 minutes by WT gDNA and MT gDNA for the MT MP-containing clinical samples (right panel) with their respective Ct values (the table). The error bars indicate the standard deviation of the means from three independent replicate experiments.

To evaluate the accuracy of PCR-PfAgo method in detecting clinical samples, oropharyngeal swab specimens were collected from clinical settings and analyzed using both the optimized PCR-PfAgo and qPCR techniques. These samples are de-identified residual clinical specimens. For MP, a total of 58 samples were evaluated: 17 negative samples and 41 positive samples. The positive samples comprised 18 simulated samples with varying concentrations of ATCC 15531 spiked into MP-negative oropharyngeal swabs and 23 clinical samples. All samples were tested after extraction. The results obtained from PCR-PfAgo were consistent with those from qPCR (as depicted in Fig. 5A). Besides, the extracted nucleic acids from all the clinical oropharyngeal swab samples underwent conventional PCR and Sanger sequencing to confirm the presence of a G at position 2063. Based on the Sanger sequencing results, the MP strains in all the clinical samples were classified as MT MP. For WT samples with an A at position 2063, simulated samples were prepared by spiking MP (ATCC 15531), which was confirmed as WT by Sanger sequencing, into oropharyngeal swabs at various concentrations. All the 18 WT MP simulated samples and 23 MT MP clinical samples were subject to MRMP (A2063G) detection. The discrimination results obtained from the PCR-PfAgo method were consistent with those derived from Sanger sequencing (Fig. 5B). Notably, even for samples with CT values close to 35, effective detections of MRMP (A2063) were achieved, demonstrating a robust discrimination effect. In addition, to reduce bias and ensure the reliability of results, we conducted blinded and randomized detection experiments to further verify the repeatability of the method. The results showed that under controlled conditions, no false positive or false negative results were observed consistently, and the relevant details are provided in Fig. S6. These validation results confirm the efficacy and reliability of the PCR-PfAgo method in detecting both MP and MRMP in actual clinical samples.

## DISCUSSIONS

In recent years, the advantages of PfAgo in *in vitro* applications have become increasingly evident. These advantages encompass programmable capability, high cleavage efficiency, rapid cleavage of target nucleic acid sequence, and independence from Protospacer Adjacent Motif (PAM) sequences—a feature distinguishing it from CRISPR systems. Consequently, PfAgo has emerged as a promising tool for pathogen nucleic acid detection. Indeed, PfAgo has been utilized in developing novel methodologies in the detection of pathogens and identifying nucleic acid mutations (31). However, despite these promising attributes, there remains a significant gap in the availability of robust methodologies that effectively leverage PfAgo for comprehensive pathogen detection.

Herein, initially, a short PCR was integrated with PfAgo for the detection of MP. Due to the selection of multi-copy repetitive elements within MP genome as the target and the high efficiency of the designed gDNA, the entire reaction was completed within 1 h. This approach achieved high sensitivity, with a limit of detection of 0.5 copies per reaction. This dual recognition and cleavage process mechanisms enhanced the specificity and accuracy of the method, thereby markedly reducing the likelihood of false positive results.

Furthermore, an important mutation, A2063G in domain V of the 23S rRNA gene in MP, is closely associated with macrolide-resistant MP. The GC content surrounding the A2063G site is unusually high (exceeding 70%), which often renders traditional PCR-based methods insensitive for detecting this mutation. By employing meticulously designed guide DNA (gDNA), we established a PfAgo-based universal mutation detection method capable of distinguishing between wild-type and mutant bases (A or G) based on differences in fluorescent signals generated by each variant. Each sample was tested using the two distinct gDNA sets: one perfectly matched (WT) and one mismatched (MT). For WT samples, the WT gDNA is fully complementary to the target, resulting in robust signal amplification. Under these conditions, the assay achieves a detection limit of 14 copies per reaction, defining its sensitivity for wild-type detection. In contrast, when MT gDNA is applied to the sample, the signal retards then resulting in no significant signal observed at 8 min, demonstrating high specificity. Conversely, for MT samples, the MT gDNA is fully matched and yields a clear signal with an LOD of 5 copies per reaction, indicating higher sensitivity for mutant detection. The WT gDNA, being mismatched, produces minimal fluorescence. The specificity in discriminating between wild-type and MT (resistant) strains is therefore achieved through dual-gDNA interrogation combined with kinetic analysis of fluorescence development. A positive signal that reaches plateau rapidly with one gDNA set—but not the other—enables confident genotyping. This temporal difference in signal kinetics ensures reliable differentiation even in the presence of low template concentrations. This approach diverges from existing PfAgo-based mutation detection methods (28, 29), which typically fail to differentiate mutation in high GC-content regions, as demonstrated in the supplementary materials (Fig. S2 and S3), where they are unable to discriminate mutant from wild-type MP at the A2063G mutation. The newly developed PCR-PfAgo method for detecting the A2063G mutation in clinical samples yielded results consistent with those obtained through the Sanger sequencing. Notably, this method was capable of identifying weakly positive samples that were below the concentration threshold detectable by PCR and standard Sanger sequencing (data not shown). These findings highlight the superior sensitivity and specificity of our approach.

Additionally, the established PfAgo method can be directly applied to detect MP in oropharyngeal swabs without the need for nucleic acid extraction. This method was tested on five oropharyngeal swab samples, successfully detecting MP in three out of the five samples. Detailed results and comparisons are provided in the supplementary materials (Fig. S7). These results demonstrate the feasibility of direct detection using unprocessed clinical specimens, but compromising sensitivity, likely due to the presence of PCR inhibitors or lower target availability in crude samples. Therefore, while promising for rapid screening, this format is currently less sensitive than the PCR-PfAgo method using extracted nucleic acid, and larger sample sizes are needed to systematically evaluate the performance of the assay on un-extracted specimens.

However, this method has several limitations. It consists of two steps: initial PCR amplification, followed by detection using the PfAgo cleavage system. A potential source of error arises during the second step when the tube is opened to add the cleavage system, as this may generate aerosols that could lead to contamination and false-positive results, and future directions may focus on developing a closed, integrated system—potentially using microfluidics or compartmentalized reactions—to combine amplification and detection in a single sealed unit. Notably, the method we employed incorporates additional base mutations into the gDNA, which may compromise the detection limit. For instance, the detection limits for differentiating WT (2063A) from MT (2063G)—14 copies per reaction and 5 copies per reaction, respectively—are significantly higher than those for MP detection (0.5 copies per reaction). Importantly, although the MP detection system targets multi-copy regions of the genome, the detection limits for WT and MT remain relatively high. The gDNA mismatch strategy used for MRMP detection introduces a slight reduction in amplification efficiency for the mutant allele, which contributes to the higher (less sensitive) limit of detection. Moreover, we observed that even when the gDNA was not fully complementary to the target sequence—such as in cases with one or more base mismatches—it could still mediate the cleavage of the target by PfAgo. Consequently, the differences between the fluorescent signals were utilized to determine the presence of the mutation A2063G mutation. To address these limitations, we are currently exploring a more sensitive method, wherein mutations generate fluorescent signals while WT sequences remain uncleaved by PfAgo. Furthermore, while the current assay demonstrates high sensitivity and specificity to detect MP and MRMP in clinical samples, it does not include an internal process control. Future clinical translation will require integration of a control system to monitor amplification efficiency and rule out false negatives due to sample inhibition or degradation, particularly in settings with variable sample quality. Nevertheless, the PCR-PfAgo system formulated here for detecting MP and MRMP with the A2063G mutation in domain V of the 23S rRNA gene has been demonstrated to sensitively and specifically identify MP and MRMP in both simulated and clinical oropharyngeal swab samples.

### Conclusions

The PCR-PfAgo system developed in this study represents a rapid, sensitive, and specific platform for the detection of MP and the identification of the prevalent A2063G macrolide resistance mutation. By integrating short PCR with PfAgo-mediated nucleic acid cleavage, the method achieves high analytical performance within an hour, enabling near-point-of-care application. Its ability to accurately distinguish resistant from susceptible strains in both simulated and clinical samples highlights its potential for guiding timely and appropriate antibiotic use in respiratory infections. This work demonstrates the feasibility of leveraging Argonaute-based systems for clinical diagnostics and provides a scalable framework for the detection of other pathogens and resistance markers. Future development could focus on multiplexing, instrument-free readout, and prospective clinical validation.

## Data Availability

The data that support the findings of this study are available from the corresponding author upon reasonable request.

## SUPPLEMENTAL MATERIAL

Supplemental figures and tables jcm.01089-25-s0001.docx Figure S1 to S7 and Tables S1 to S4.

ASM does not own the copyrights to Supplemental Material that may be linked to, or accessed through, an article. The authors have granted ASM a non-exclusive, world-wide license to publish the Supplemental Material files. Please contact the corresponding author directly for reuse.
