# Supplementary material for: A novel Pyrococcus furiosus argonaute-based method for rapid and sensitive detection of Mycoplasma pneumoniae and a macrolide-resistance-related mutation
Source: J Clin Microbiol. 2025 Dec 18;64(1):e01089-25. doi: 10.1128/jcm.01089-25 (PMC12802221; doi:10.1128/jcm.01089-25)
Supplement: Supplemental figures and tables — Figure S1 to S7 and Tables S1 to S4. [file jcm.01089-25-s0001.docx]

**Supplementary Materials**

**A novel *Pyrococcus furiosus* Argonaute-based method for Rapid and Sensitive detection of *Mycoplasma Pneumoniae* and a Macrolide-resistance-related mutation**

Yun Zhang ^a, #^, Chunhui Huo ^a, #^, Tao Zhang ^d^, Qian Liu ^a^, Ping He ^b, c^, Jiansen Du ^d^, Dongyan Xiong ^b, c^, Hongping Wei ^b, c, *^, Junping Yu ^a, b, c*^

^a^ School of Medical Technology, Xinxiang Medical University, Xinxiang 453003, PR China

^b^ CAS Key Laboratory of Special Pathogens and Biosafety, Center for Emerging Infectious Diseases, Wuhan Institute of Virology, Chinese Academy of Sciences, Wuhan 430071, PR China

^c^ University of Chinese Academy of Sciences, Beijing 100049, PR China

^d^ Qingdao International Travel Health care Center，Qingdao Customs, Qingdao 266000, PR China

^*^Corresponding authors:

Junping Yu, Email: [yujp@wh.iov.cn](mailto:yujp@wh.iov.cn); Tel: +86 27 87998263.

Hongping Wei, Email: [hpwei@wh.iov.cn](mailto:hpwei@wh.iov.cn); Tel: +86 27 87998873. Gold Science & Technology Park, Jinlong Street, No 262, Jiangxia District, Wuhan, Hubei province, 430207, China

^#^These authors contribute equally.


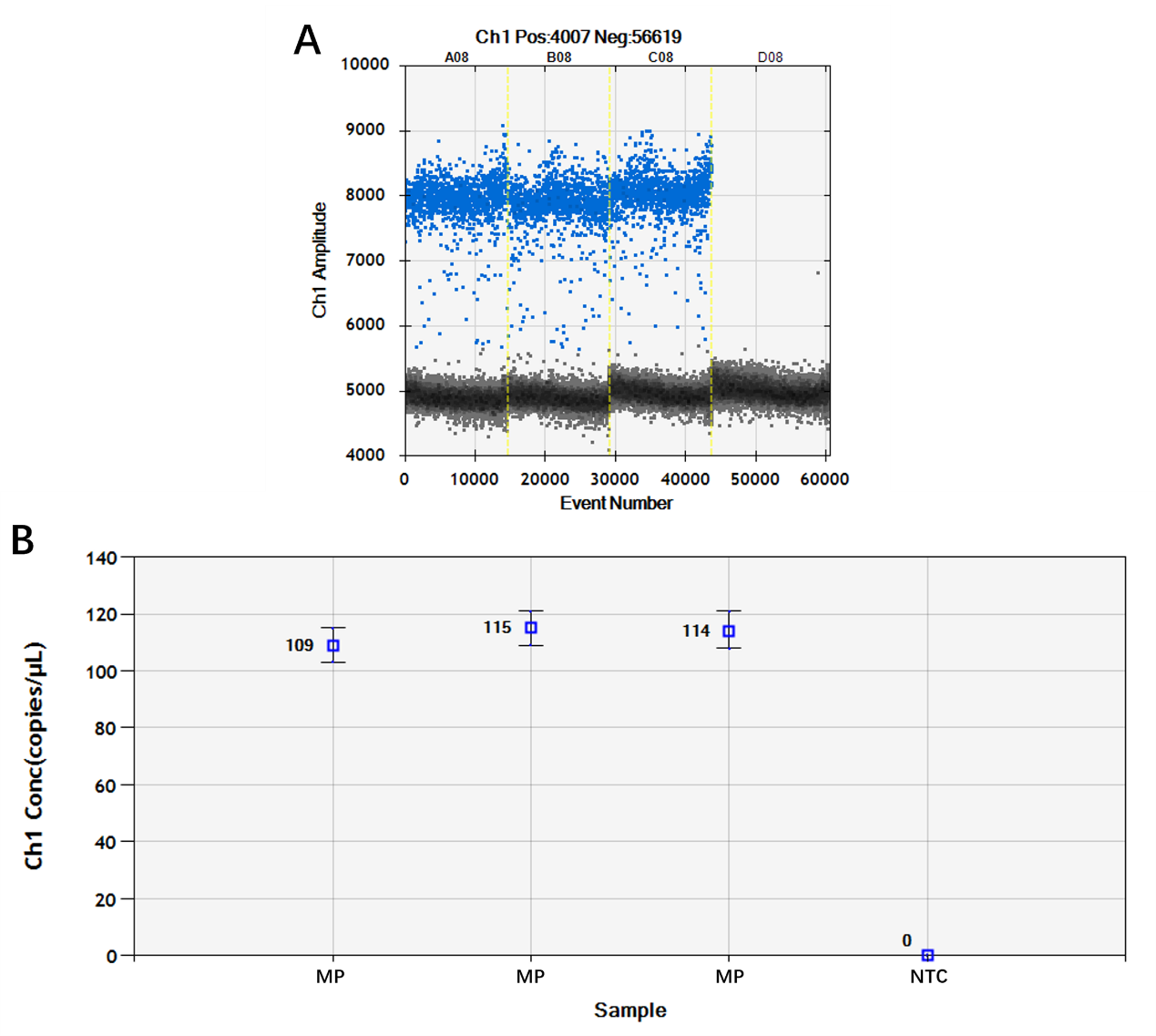


**
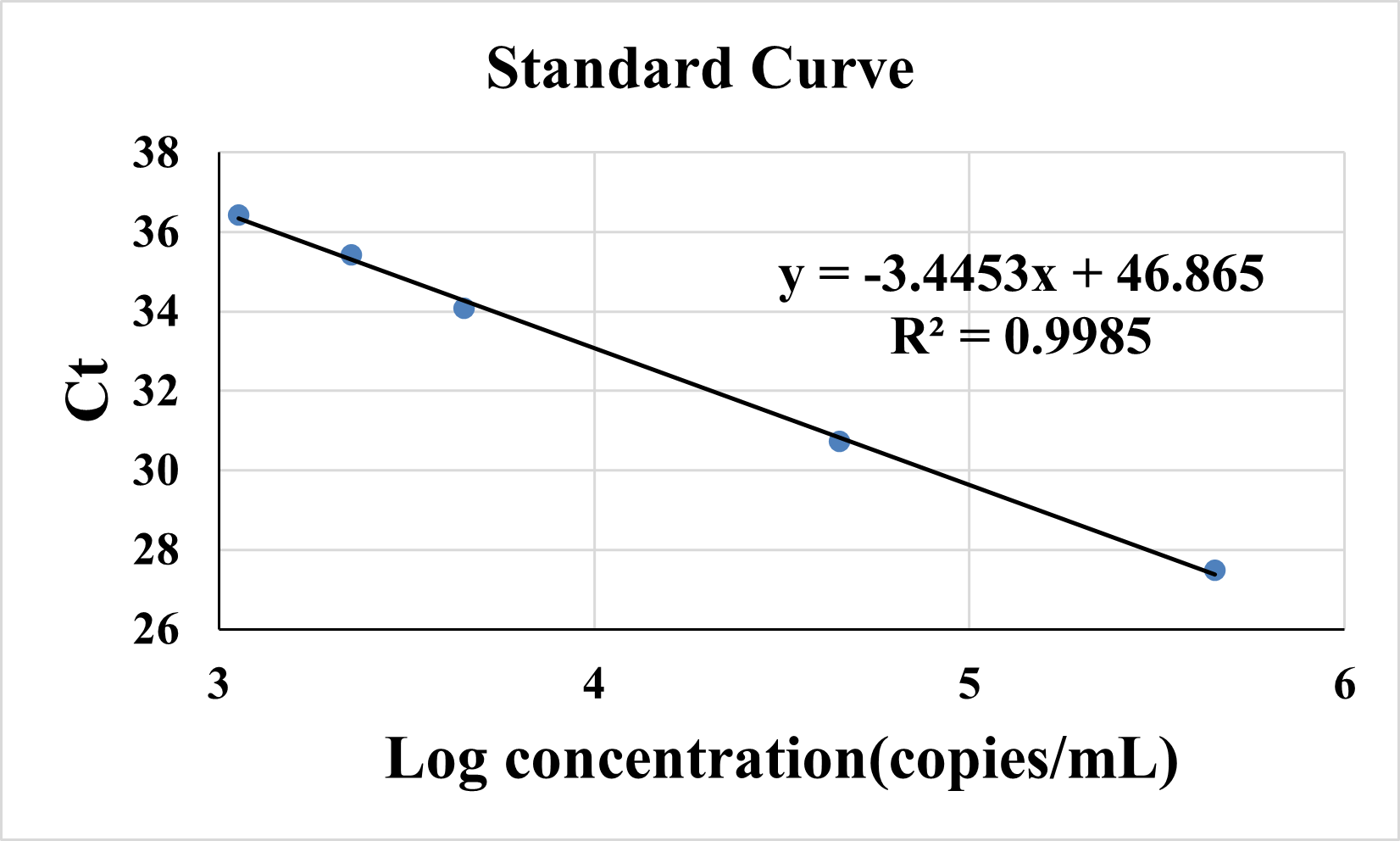
**

**C**

**Figure S1.** Determination of the copy number of the extracted DNA using ddPCR. (A)Scatter plot of ddPCR results for *Mycoplasma pneumoniae* (MP). (B)Copy concentration in a 20 μL ddPCR reaction mixture. Three replicate experiments were conducted, along with one negative control. The DNA was extracted from inactivated MP samples. (C) Standard curve fitted using Ct values and the logarithm of DNA concentration [Log10(copies/mL)].


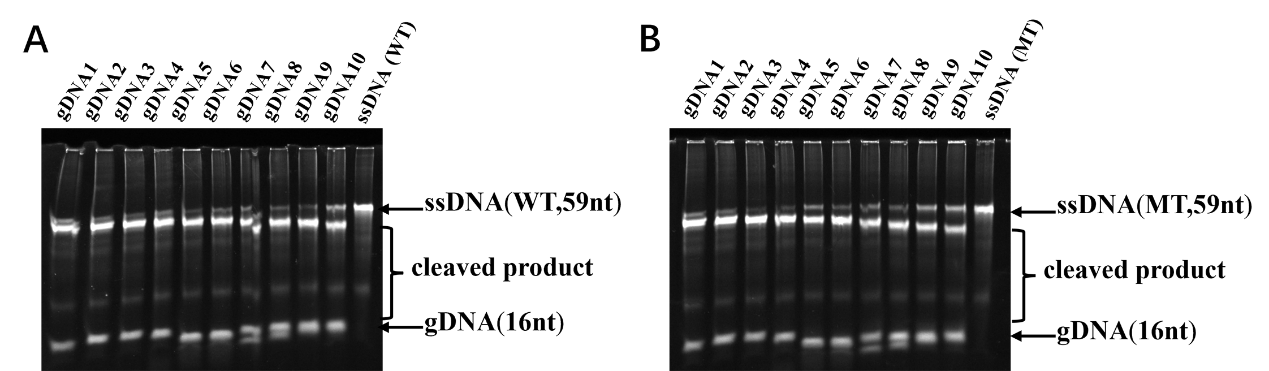


**Figure S2.** Screening of gDNA for macrolide-resistant *Mycoplasma pneumoniae* (MRMP) Detection-1. Urea-PAGE images illustrate the PfAgo-mediated cleavage of ssDNA templates (derived from 23S rRNA) guided by gDNA1 to gDNA10. (A) The ssDNA template corresponds to the wild type (WT) sequence. (B) The ssDNA template correspond to the mutant type (MT) sequence.


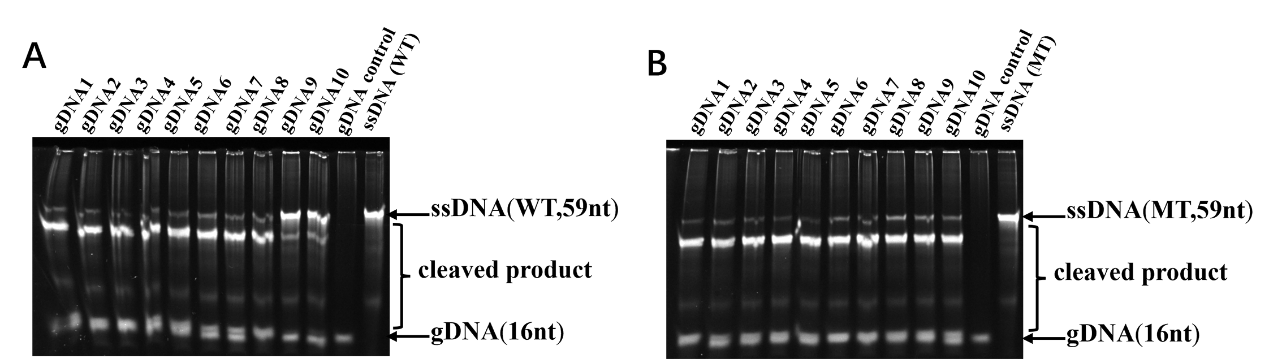


**Figure S3.** Screening of gDNA for MRMP Detection-2. Urea-PAGE images show PfAgo-mediated cleavage of ssDNA templates (derived from 23S rRNA) guided by gDNA0 and gDNA1 to gDNA10. (A) The ssDNA template corresponds to the wild-type (WT) sequence. (B) The ssDNA template corresponds to the mutant-type (MT) sequence.


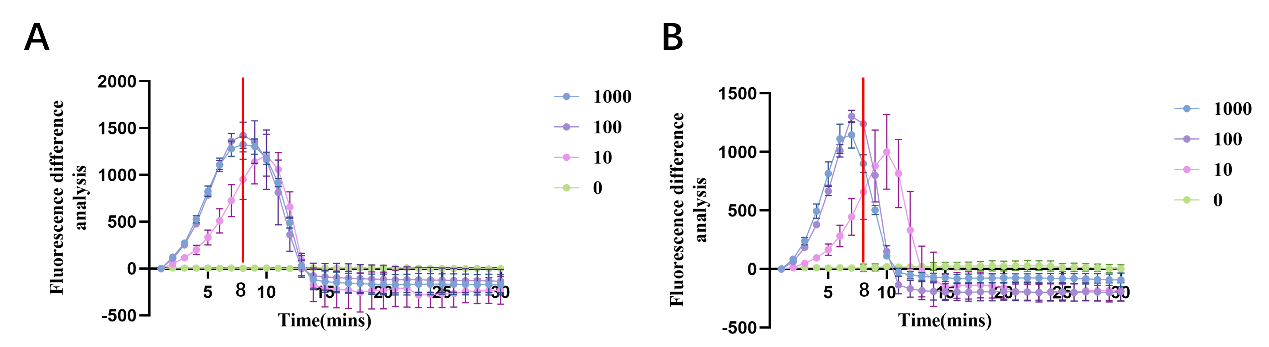


**Figure S4.** For wild-type and mutant samples at high, medium, and low concentrations (copies/reaction), two sets of gDNA were used for detection. The background fluorescence values were subtracted, and the changes in the difference between the fluorescence values guided by the two sets of gDNA over time were quantified. (A) Mutant-type samples; (B) Wild-type samples. Error bars represent the standard deviation of three replicate experiments.


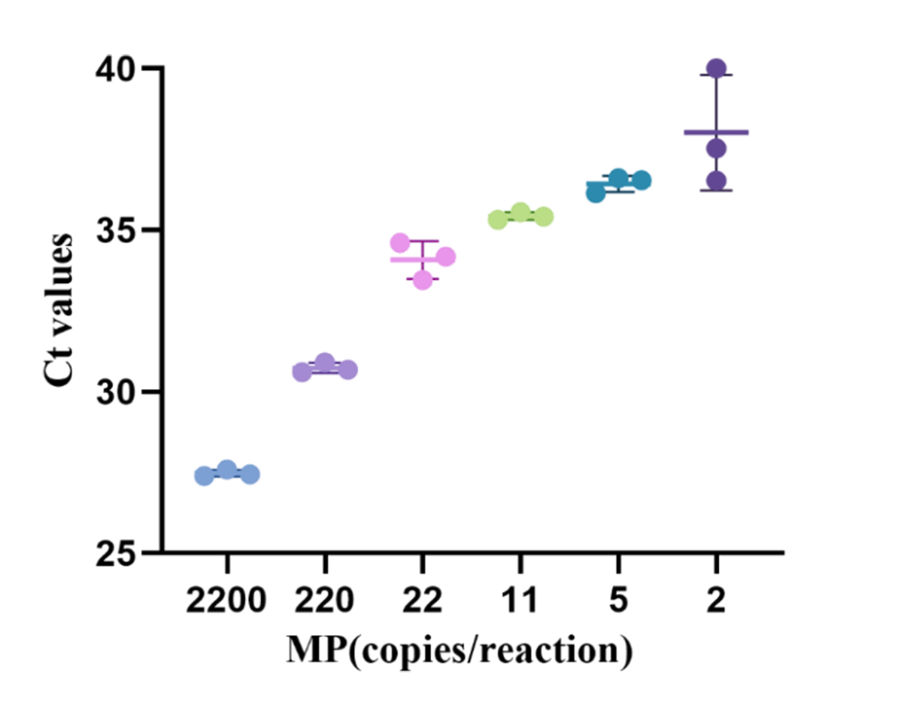


**Figure S5.** Detection limit of a commercial qPCR kit (Q112) for MP. Three replicate experiments were performed for each concentration. **Note:** CT values marked as “NA” were recorded as 40.


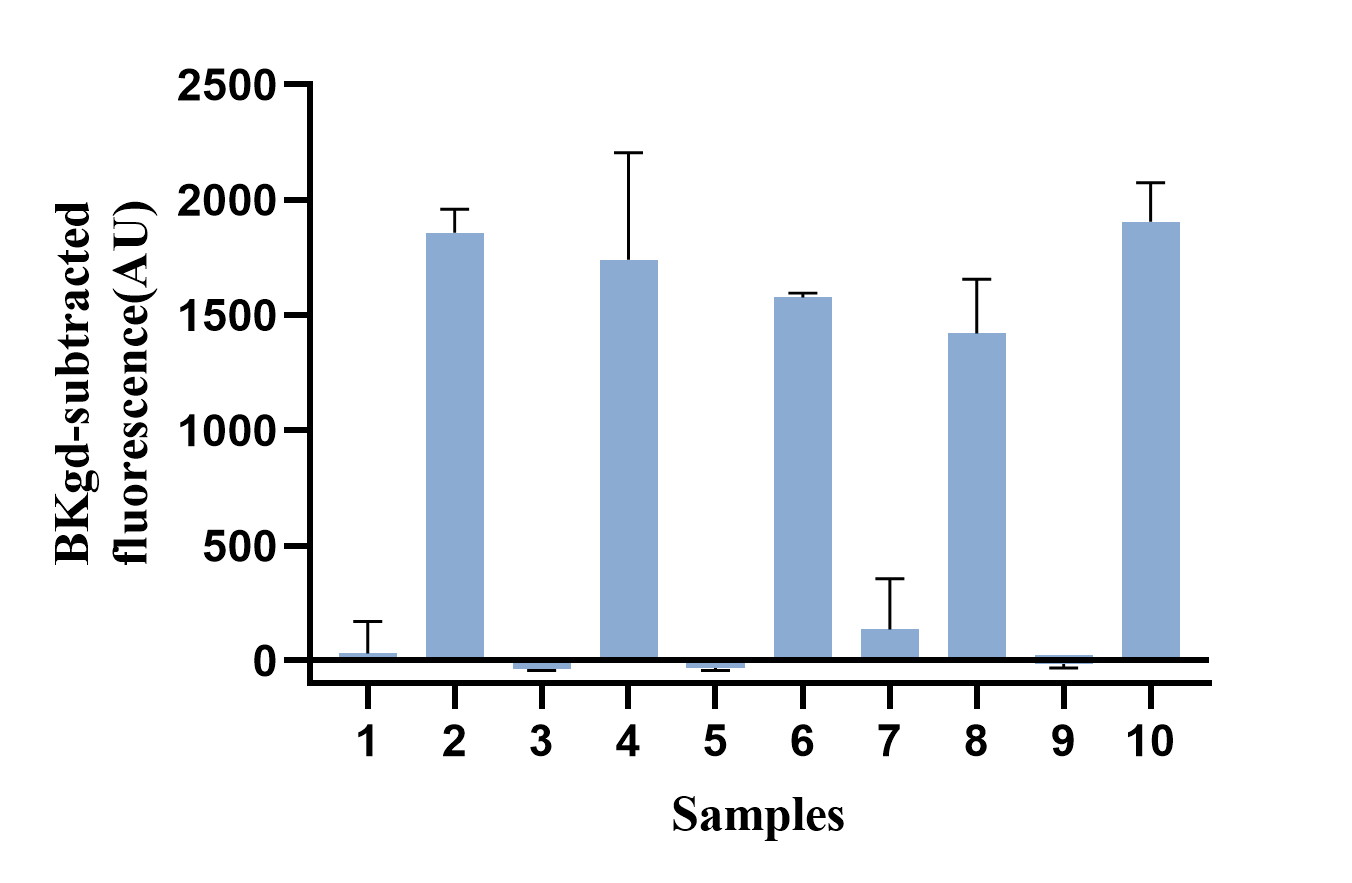


**Figure S6.** Blinded and randomized testing of samples. Samples 1, 3, 5, and 9 were negative controls, while samples 2, 4, 6, 8, and 10 were positive. The randomized blinded testing performed by the operators showed no false-positive or false-negative results.


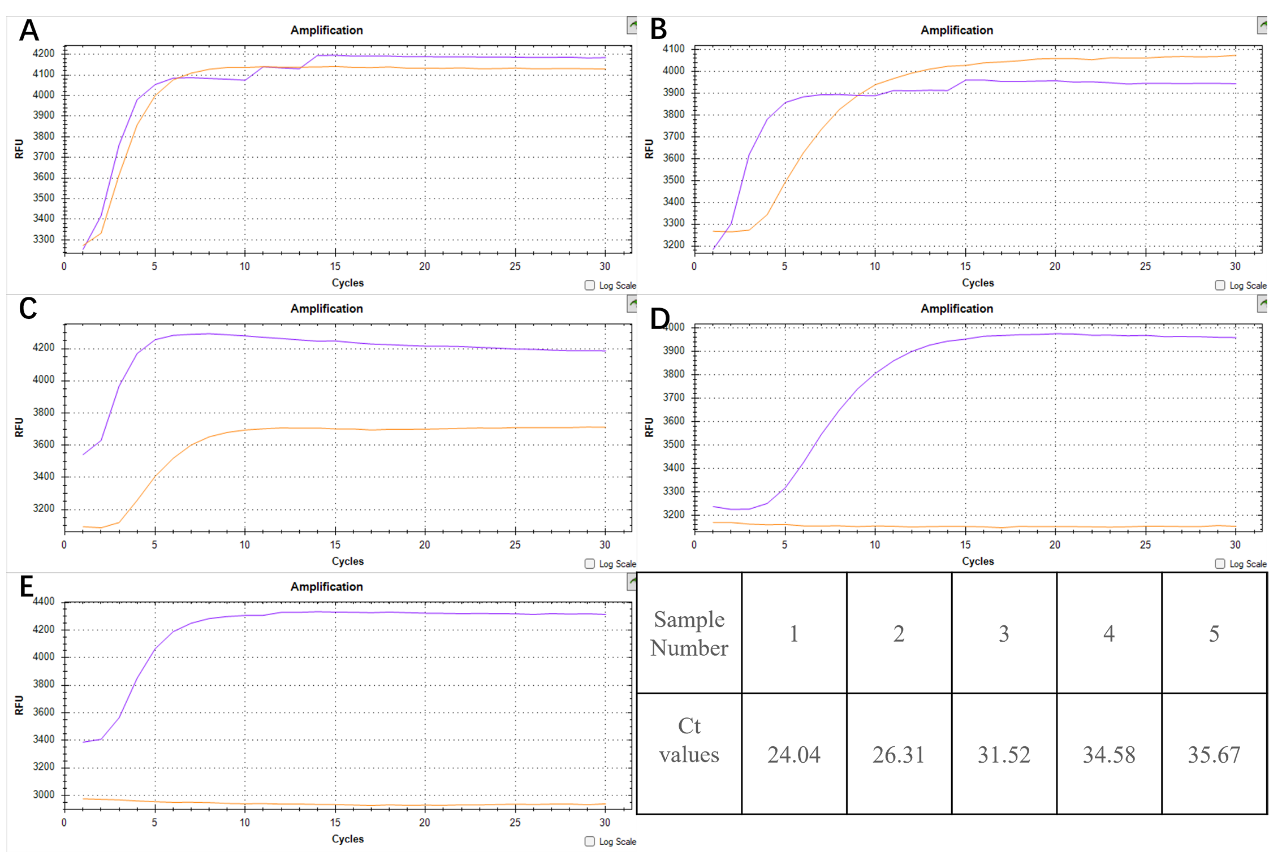


**Figure S7.** Comparison of nucleic acid test results from throat swab samples with and without nucleic acid extraction. The serial numbers for the samples corresponding to Figures (A), (B), (C), (D), and (E) were 1, 2, 3, 4, and 5, respectively. The Ct values for each sample are provided in the table. The yellow lines represent samples that did not undergo nucleic acid extraction, whereas the purple lines depict samples that underwent nucleic acid extraction.

**Table S1.** Sequences of the PCR detection templates for MP and MRMP.

| RepMp1 | AACTCTTTACGCGTTACGTATTCCGTGTTCACTGGTATAACCGGTTTGTTAAGTTTAAATTGTCTGTTGAATTTGCCAGAGAATTCCACACTGTTGTT |
| --- | --- |
| 23S rRNA (WT) | ATCCAGGTACGGGTGAAGACACCCGTTAGGCGCAACGGGACGGAAAGACCCCGTGAAGCTTTACTGTAGCTTAATATTGATCAGGAC |
| 23S rRNA (MT) | ATCCAGGTACGGGTGAAGACACCCGTTAGGCGCAACGGGACGGGAAGACCCCGTGAAGCTTTACTGTAGCTTAATATTGATCAGGAC |

**Table S2.** Sequences used in the gDNA screening process for MRMP.

| Name | Sequence (5'-3') |
| --- | --- |
| gDNA1(23S rRNA) | GAAGACCCCGTGAAGC |
| gDNA2(23S rRNA) | GGAAGACCCCGTGAAG |
| gDNA3(23S rRNA) | GGGAAGACCCCGTGAA |
| gDNA4(23S rRNA) | CGGGAAGACCCCGTGA |
| gDNA5(23S rRNA) | ACGGGAAGACCCCGTG |
| gDNA6(23S rRNA) | GACGGGAAGACCCCGT |
| gDNA7(23S rRNA) | GGACGGGAAGACCCCG |
| gDNA8(23S rRNA) | GGGACGGGAAGACCCC |
| gDNA9(23S rRNA) | CGGGACGGGAAGACCC |
| gDNA10(23S rRNA) | ACGGGACGGGAAGACC |
| ssDNA (23S rRNA-WT) | GCTTCACGGGGTCTTTCCGTCCCGTTGCGCCTAACGGGTGTCTTCACCCGTACCTGGAT |
| ssDNA (23S rRNA-MT) | GCTTCACGGGGTCTTCCCGTCCCGTTGCGCCTAACGGGTGTCTTCACCCGTACCTGGAT |

**Table S3.** Sequences used in the gDNA screening process for MRMP.

| Name | Sequence (5'-3') |
| --- | --- |
| gDNA0(23S rRNA) | TGAAGACACCCGTTAG |
| gDNA1(23S rRNA) | TACGGGACGGGAAGAC |
| gDNA2(23S rRNA) | ATCGGGACGGGAAGAC |
| gDNA3(23S rRNA) | AAGGGGACGGGAAGAC |
| gDNA4(23S rRNA) | AACCGGACGGGAAGAC |
| gDNA5(23S rRNA) | AACGCGACGGGAAGAC |
| gDNA6(23S rRNA) | AACGGCACGGGAAGAC |
| gDNA7(23S rRNA) | AACGGGTCGGGAAGAC |
| gDNA8(23S rRNA) | AACGGGAGGGGAAGAC |
| gDNA9(23S rRNA) | AACGGGACCGGAAGAC |
| gDNA10(23S rRNA) | AACGGGACGCGAAGAC |
| ssDNA (23S rRNA-WT) | GCTTCACGGGGTCTTTCCGTCCCGTTGCGCCTAACGGGTGTCTTCACCCGTACCTGGAT |
| ssDNA (23S rRNA-MT) | GCTTCACGGGGTCTTCCCGTCCCGTTGCGCCTAACGGGTGTCTTCACCCGTACCTGGAT |

**Table S4.** Sequences of the primer sequences for *Ureaplasma urealyticum*.

| Name | Sequence (5'-3') |
| --- | --- |
| UreaPlasma-F | GGAACTGGAGGACATAAC^*^ |
| UreaPlasma-R | TTACGACAGGAACTGATAC |

* The sequences were referred to a research work (Krzysztoń-Russjan J, *et al*. Development of New PCR Assay with SYBR Green I for Detection of Mycoplasma, Acholeplasma, and Ureaplasma sp. in Cell Cultures. Diagnostics (Basel). 2021;11(5):876. doi: 10.3390/diagnostics11050876.)

**qPCR System and Protocol for detection of *Ureaplasma urealyticum*.**

ChamQ Universal SYBR qPCR Master Mix (Q711, Vazyme, Nanjing, China) was used to prepare the qPCR detection system, which consisted of 5 μL of template RNA, 10 μL of Mix, 0.2 μM of forward primer, 0.2 μM of reverse primer, and nuclease-free water, bringing the final volume to 20 μL. The qPCR reaction is conducted on CFX96 Real-Time PCR instrument (Bio-rad, CA, USA), which involves an initial predenaturation step at 95°C for 30 s and subsequent 40 cycles of denaturation at 95°C for 10 s and annealing at 60°C for 30 s, during which fluorescence signals are collected.
